# Changes in Health Care Access and Preventive Health Screenings by Race and Ethnicity

**DOI:** 10.1001/jamahealthforum.2023.5058

**Published:** 2024-02-02

**Authors:** Christopher Alba, ZhaoNian Zheng, Rishi K. Wadhera

**Affiliations:** 1 Richard A. and Susan F. Smith Center for Outcomes Research, Beth Israel Deaconess Medical Center, Boston, Massachusetts; 2 Harvard Medical School, Boston, Massachusetts

## Abstract

**Importance:**

The COVID-19 pandemic led to unprecedented disruptions in health care. Little is known about whether health care access and preventive health screenings among US adults have recovered to prepandemic levels, and how patterns varied by race and ethnicity.

**Objective:**

To evaluate health care access and preventive health screenings among eligible US adults in 2021 and 2022 compared with prepandemic year 2019, overall and by race and ethnicity.

**Design, Setting, and Participants:**

This cross-sectional study used data from US adults aged 18 years or older who participated in the National Health Interview Survey in 2021 and 2022. Survey weights provided by the National Health Interview Survey were used to generate nationally representative estimates. Data were analyzed from May 23 to November 13, 2023.

**Main Outcomes and Measures:**

Measures of health care access included the proportion of adults with a usual place for care, those with a wellness visit, and those who delayed or did not receive medical care due to cost within the past year. Preventive health screening measures included eligible adults who received blood pressure, cholesterol, or blood glucose screening within the past year (2021), as well as colorectal, cervical, breast, and prostate cancer screenings based on US Preventive Services Task Force guidelines.

**Results:**

The unweighted study population included 89 130 US adults. The weighted population included 51.6% females; 16.8% Hispanic, 5.9% non-Hispanic Asian (hereafter, Asian), 11.8% non-Hispanic Black (hereafter, Black), 62.8% non-Hispanic White (hereafter, White) individuals; and 2.9% individuals of other races and ethnicities (including American Indian, Alaska Native, Native Hawaiian or other Pacific Islander, or multiracial). After adjusting for age and sex, having a usual place for health care did not differ among adults in 2021 or 2022 vs 2019 (adjusted rate ratio [ARR] for each year, 1.00; 95% CI, 0.99-1.01). However, fewer participants had wellness visits in 2022 compared with 2019 (ARR, 0.98; 95% CI, 0.97-0.99), with the most pronounced decline among Asian adults (ARR, 0.95; 95% CI, 0.92-0.98). In addition, adults were less likely to delay medical care (ARR, 0.79; 95% CI, 0.73-0.87) or to not receive care (ARR, 0.76; 95% CI, 0.69-0.83) due to cost in 2022 vs 2019. Preventive health screenings in 2021 remained below 2019 levels (blood pressure: ARR, 0.95 [95% CI, 0.94-0.96]; blood glucose: ARR, 0.95 [95% CI, 0.93-0.96]; and cholesterol: ARR, 0.93 [95% CI, 0.92-0.94]). Eligible adults were also significantly less likely to receive colorectal cancer screening (ARR, 0.88; 95% CI, 0.81-0.94), cervical cancer screening (ARR, 0.86; 95% CI, 0.83-0.89), breast cancer screening (ARR, 0.93; 95% CI, 0.90-0.97), and prostate cancer screening (ARR, 0.86 [0.78-0.94]) in 2021 vs 2019. Asian adults experienced the largest relative decreases across most preventive screenings, while Black and Hispanic adults experienced large declines in colorectal cancer screening (ARR, 0.78; 95% CI, 0.67-0.91) and breast cancer screening (ARR, 0.83; 95% CI, 0.75-0.91), respectively. Differences in preventive screening rates across years persisted after additional adjustment for socioeconomic factors (income, employment status, and insurance coverage).

**Conclusions and Relevance:**

Results of this cohort study suggest that, in the US, wellness visits and preventive health screenings have not returned to prepandemic levels. These findings support the need for public health efforts to increase the use of preventive health screenings among eligible US adults.

## Key Points

**Question:**

Have health care access and use of preventive health screenings returned to prepandemic levels among US adults, and are there differences in access and screenings by race and ethnicity?

**Findings:**

In this cross-sectional study of 89 130 US adults, there were fewer outpatient wellness visits in 2022 compared with 2019. Screening rates for blood pressure, cholesterol, blood glucose, and common cancers were lower in 2021 vs 2019, and varied across racial and ethnic groups, with Asian adults experiencing the most pronounced declines.

**Meaning:**

Findings of this study suggest that wellness visits and preventive health screenings have not returned to prepandemic levels, supporting the need for efforts to increase preventive health screenings among US adults.

## Teaser

This cross-sectional study examines changes in wellness visits and preventive health screening before and after the COVID-19 pandemic according to race and ethnicity.

## Introduction

In the US, the COVID-19 pandemic onset led to unprecedented disruptions in health care.^1,2,3^ Primary care visits declined markedly during the first few months of the pandemic, and 1 in 5 adults delayed or did not receive necessary medical care.^2^ As a result, guideline-recommended screening for medical conditions, such as hypertension, diabetes, and cancer, decreased sharply during the first few months of 2020.^4,5,6,7^

Clinicians, health system leaders, and policymakers have raised concerns that persistent disruptions in health care access and preventive health screenings could have long-term public health consequences.^8,9^ However, little is known about whether health care access (eg, usual place for care, wellness visits) have returned to prepandemic levels in 2022. In addition, although prior analyses have evaluated pandemic-related changes in the receipt of some types of preventive screenings,^10,11,12^ they have largely focused on older age groups and have not assessed the extent to which socioeconomic factors, such as increased unemployment and economic loss during the pandemic, contributed to observed changes. On one hand, the launch of local and national initiatives as well as policies that bolstered the safety net may have helped increase health care access and preventive screenings as the pandemic continued, particularly among racial and ethnic minority groups.^13^ However, outpatient visits in the US remained below prepandemic levels through 2022, which has raised concern about ongoing disruptions in care.^14^ Understanding whether health care access and preventive screenings have returned to prepandemic levels has critically important public health implications and can inform public health strategies to help mitigate the consequences related to the COVID-19 pandemic.

Therefore, in this study, we evaluated changes in measures of health care access and preventive health screenings among US adults in 2021 and 2022 compared with prepandemic levels in 2019 to ascertain whether these patterns differed by race and ethnicity. We also assessed whether changes in socioeconomic factors (income, employment status, insurance coverage) were a factor in any observed differences in access and screenings.

## Methods

### Study Population

We used data from the National Health and Interview Survey (NHIS), a nationally representative, cross-sectional household interview survey of noninstitutionalized US adults conducted by the National Center for Health Statistics.^15,16,17^ The NHIS uses a geographically clustered technique to select a random sample of households from 50 US states and the District of Columbia, and adults aged 18 years or older are randomly selected from households for a detailed interview. We included data from the 2019, 2021, and 2022 NHIS cycles to evaluate outcomes before and during the pandemic, while 2020 data were excluded because the pandemic’s onset created challenges for performing the in-person survey.^18^ Measures of health care access are collected annually by NHIS, while information on preventive screenings is collected every other year (ie, 2019 and 2021). This cross-sectional study is a secondary analysis of publicly available deidentified data; therefore, it was exempt from review and the informed consent requirement in accordance with Beth Israel Deaconess Medical Center policy. We followed the Strengthening the Reporting of Observational Studies in Epidemiology (STROBE) reporting guideline.

The NHIS collects information on baseline sociodemographics and clinical comorbidities. Race and ethnicity were self-reported and included Hispanic, non-Hispanic Asian (hereafter, Asian), non-Hispanic Black or African American (hereafter, Black), and non-Hispanic White (hereafter, White). Due to small sample size, American Indian or Alaska Native, Native Hawaiian or other Pacific Islander, multiracial, and multiethnic groups were combined into a single category for this analysis as other race and ethnicity.

### Outcomes

We examined measures of health care access and preventive health screenings. Measures of health care access included the proportion of adults having a usual place of care, having a wellness visit within the past year (including physical examinations and general-purpose checkups), delaying medical care due to cost, and not receiving medical care due to cost during the past year. Preventive health screening measures included the proportion of eligible adults receiving a blood pressure, cholesterol, or blood glucose screening (inclusive of screenings performed outside of a traditional wellness visit) as well as colorectal, cervical, breast, and prostate cancer screenings within the past year.

All adults were considered eligible for blood pressure and cholesterol screenings, while adults without a diagnosis of diabetes were considered eligible for blood glucose screening. We used the US Preventive Services Task Force guidelines to determine eligibility for cancer screenings as of 2019, which included all adults aged 50 to 75 years for colorectal cancer screening (colonoscopy or sigmoidoscopy), female adults aged 21 to 65 years for cervical cancer screening (Papanicolaou smear or human papillomavirus test), female adults aged 50 to 74 years for breast cancer screening (mammography), and male adults aged 55 to 69 years for prostate cancer screening (prostate-specific antigen test).^19,20,21,22^

### Statistical Analysis

We first compared baseline sociodemographic and clinical characteristics of US adults in the prepandemic (2019) and postpandemic (combined 2021 and 2022) periods using the *t* test for continuous outcomes and the χ^2^ test for categorical outcomes. The observed proportion of adults reporting access measures and preventive screenings were characterized for each study year available, both for the overall cohort and stratified by race and ethnicity.

We then fit survey-weighted multivariable Poisson regression models to compare each outcome of interest, with results reported as adjusted rate ratios (ARRs) with 95% CIs. For measures of health care access, we compared data for 2021 with those for 2019 and, separately, data for 2022 with those for 2019 after adjusting for age and sex. For preventive screenings, we compared 2021 data with 2019 data after adjusting for age and sex. We did not adjust for other covariates (eg, income, employment status, insurance coverage) in the main analyses, as our goal was to characterize changes in access and preventive screenings. Next, to evaluate whether pandemic-related changes in socioeconomic factors (income, employment status, insurance coverage) were associated with differences in age- and sex-adjusted outcomes across years, we included these variables in the main models. All models included an interaction term for race and ethnicity × year to evaluate whether there were differential changes in outcomes across racial and ethnic subgroups. For all analyses, survey weights provided by NHIS based on probability of respondent selection and adjusted for nonresponse patterns (considering factors such as age, sex, race and ethnicity, educational attainment, and Metropolitan Statistical Area) were used to generate nationally representative estimates. The 95% CIs were estimated using NHIS’s stratum and primary sampling unit variables to account for the complex, cluster-stratified design of the national survey.

As an additional analysis, we assessed whether eligible adults were up to date on their preventive screenings in 2021 vs 2019. Adults were considered up to date on screenings if they received a blood pressure screening in the past year, cholesterol screening in the past 5 years, blood glucose screening in the past 3 years, colonoscopy in the past 10 years or sigmoidoscopy in the past 5 years, Papanicolaou smear in the past 3 years or human papillomavirus test in the past 5 years, mammography in the past 2 years, and prostate-specific antigen testing in the past 2 years.

A 2-sided *P* < .05 was used to determine statistical significance. Data analysis was conducted from May 23 to November 13, 2023, using SAS Enterprise Guide, version 7.15 (SAS Institute Inc).

## Results

The overall study sample included 89 130 adults over the 3 study years. The weighted population included 51.6% females; 16.8% Hispanic, 5.9% Asian, 11.8% Black, 62.8% White individuals; and 2.9% individuals of other races and ethnicities (including American Indian, Alaska Native, Native Hawaiian or other Pacific Islander, or multiracial). Baseline characteristics of survey participants are shown in Table 1. Among the weighted population, adults were slightly younger in 2019 compared with 2021 to 2022 (mean [SD] age, 47.4 [0.2] years vs 48.1 [0.1] years). The proportion of adults with health insurance coverage increased from 88.3% in 2019 to 90.1% in the 2021 to 2022 period (*P* < .001), while the proportion who were employed decreased from 64.6% to 62.9% (*P* < .001). The distribution of clinical comorbidities for each year is also shown in Table 1.

**Table 1.** Baseline Characteristics of US Adults in the National Health Interview Survey Before (2019) and During (2021-2022) the COVID-19 Pandemic

Characteristic	Weighted proportion of adults, % (95% CI)		*P* value
	2019 (n = 31 997)	2021-2022 (n = 57 133)	
**Demographics**			
Age, mean (SD), y	47.4 (0.2)	48.1 (0.11)	.02
Sex^a^			
Female	51.7 (51.0-52.4)	51.5 (51.0-52.0)	.65
Male	48.3 (47.6-49.0)	48.5 (48.0-49.0)	
Race and ethnicity			
Asian	5.9 (5.4-6.4)	6.0 (5.5-6.5)	.47
Black	11.8 (10.9-12.6)	11.8 (11.0-12.6)	
Hispanic	16.5 (15.2-17.8)	17.1 (15.8-18.3)	
White	63.2 (61.7-64.8)	62.4 (60.9-63.9)	
Other^b^	2.6 (2.1-3.1)	2.7 (2.3-3.2)	
Insurance coverage^c^	88.3 (87.7-89.0)	90.1 (89.6-90.6)	<.001
Employed^d^	64.6 (63.8-65.3)	62.9 (62.2-63.5)	<.001
Family income as percent of FPL			
<100% FPL	11.2 (10.6-11.8)	9.8 (9.3-10.3)	<.001
100%-199% FPL	18.7 (18.0-19.3)	17.6 (17.1-18.2)	
200%-299% FPL	17.0 (16.4-17.5)	16.3 (15.9-16.7)	
>300% FPL	53.1 (52.0-54.2)	56.3 (55.3-57.2)	
**Clinical comorbidities**			
Anxiety	14.1 (13.6-14.7)	17.1 (16.6-17.5)	<.001
Asthma	13.5 (13.0-13.9)	14.1 (13.7-14.5)	.04
Cancer	9.5 (9.1-9.9)	9.7 (9.4-10.0)	.43
COPD, emphysema, or bronchitis	4.6 (4.3-4.9)	4.6 (4.4-4.8)	.94
Coronary heart disease	4.6 (4.3-4.9)	4.9 (4.7-5.1)	.04
Depression	15.8 (15.2-16.3)	17.8 (17.3-18.2)	<.001
Diabetes	9.3 (9.0-9.7)	9.6 (9.3-9.9)	.28
Hyperlipidemia	24.9 (24.2-25.5)	27.1 (26.6-27.6)	<.001
Hypertension	31.7 (30.9-32.4)	31.7 (31.2-32.3)	.81
Myocardial infarction	3.1 (2.9-3.4)	3.0 (2.8-3.2)	.29
Obesity^e^	32.1 (31.4-32.8)	33.0 (32.4-33.6)	.04
Stroke	3.1 (2.9-3.3)	2.8 (2.7-3.0)	.05

Abbreviations: COPD, chronic obstructive pulmonary disease; FPL, federal poverty level.

^a^ Sex self-reported as a binary choice between male or female. Fewer than 0.01% of respondents reported do not know or refused to answer.

^b^ Includes American Indian or Alaska Native, Native Hawaiian or other Pacific Islander, or multiracial.

^c^ Insurance coverage includes those who have private insurance, Medicare, Medicaid, state Children’s Health Insurance Program, a state-sponsored health plan, other government programs, or a military health plan.

^d^ A person was considered employed if they reported working in the past week, performing seasonal or contract work, or working at a job or business but not for pay. In 2019, people who reported any seasonal or contract work were considered employed for 2019; only those who reported seasonal or contract work in the past 12 months were considered employed for 2021 and 2022.

^e^ Defined as a body mass index ≥30 (calculated as weight in kilograms divided by height in meters squared).

### Health Care Access

Measures of health care access are shown in Table 2. After adjustment for age and sex, there was no change in having a usual place for care in 2021 (ARR, 1.00; 95% CI, 0.99-0.01) and 2022 (ARR, 1.00; 95% CI, 0.99-1.01) compared with 2019, and these patterns were similar across racial and ethnic subgroups. In contrast, having a wellness visit within the past year was less common in 2021 (compared with 2019) for all adults (ARR, 0.96; 95% CI, 0.95-0.97), a change most pronounced in Asian adults compared with White adults (ARR, 0.86 [95% CI, 0.83-0.90]; *P* for interaction <.001). Having a wellness visit within the past year continued to remain below prepandemic levels in 2022 (ARR, 0.98; 95% CI, 0.97-0.99]), particularly among Asian adults (compared with White adults: ARR, 0.95 [95% CI, 0.92-0.98]; *P* for interaction = .02), while Black, Hispanic, and White adults had rates similar to those in the prepandemic period. Fewer adults reported delaying medical care due to cost (ARR, 0.79; 95% CI, 0.73-0.87) and not receiving care due to cost (ARR, 0.76; 95% CI, 0.69-0.83) in 2022 compared with 2019, overall and among Black, Hispanic, and White adults.

**Table 2.** Measures of Health Care Access for US Adults in 2021 and 2022 Compared With 2019

Measure	2019 Weighted estimate, % (95% CI)	2021 Weighted estimate, % (95% CI)	Adjusted rate ratio for 2021 vs 2019 (95% CI)^a^	*P* value for interaction^b^	2022 Weighted estimate, % (95% CI)	Adjusted rate ratio for 2022 vs 2019 (95% CI)^a^	*P* value for interaction^b^
**Have a usual place for care**							
Asian	90.2 (88.5-92.0)	89.0 (87.3-90.8)	0.99 (0.96-1.01)	.28	89.4 (87.6-91.1)	0.99 (0.96-1.01)	.35
Black	89.9 (88.5-91.3)	90.0 (88.5-91.5)	1.00 (0.98-1.02)	.84	90.1 (88.6-91.7)	1.00 (0.98-1.02)	.90
Hispanic	83.3 (81.6-84.9)	84.5 (83.1-86.0)	1.01 (0.99-1.04)	.26	82.6 (81.0-84.3)	0.99 (0.97-1.02)	.56
White	91.0 (90.4-91.5)	91.1 (90.5-91.6)	1.00 (0.99-1.01)	[Reference]	91.0 (90.4-91.6)	1.00 (0.99-1.01)	[Reference]
Other^c^	88.1 (84.7-91.5)	86.9 (83.0-90.7)	0.99 (0.95-1.04)	.71	89.0 (85.6-92.3)	1.02 (0.98-1.06)	.40
Overall	89.4 (88.9-90.0)	89.6 (89.1-90.1)	1.00 (0.99-1.01)	NA	89.3 (88.7-89.9)	1.00 (0.99-1.01)	NA
**Had a wellness visit in past year^d^**							
Asian	86.1 (84.0-88.2)	74.4 (71.8-77.0)	0.86 (0.83-0.90)	<.001	81.9 (79.7-84.2)	0.95 (0.92-0.98)	.02
Black	87.1 (85.5-88.7)	86.0 (84.3-87.6)	0.98 (0.96-1.01)	.36	84.7 (83.0-86.4)	0.97 (0.94-1.00)	.19
Hispanic	77.0 (75.2-78.8)	74.8 (73.0-76.6)	0.97 (0.94-1.00)	.97	76.0 (74.4-77.7)	0.99 (0.96-1.01)	.85
White	85.9 (85.2-86.5)	83.4 (82.7-84.2)	0.97 (0.96-0.98)	[Reference]	85.0 (84.3-85.8)	0.99 (0.98-1.00)	[Reference]
Other^c^	82.0 (78.0-86.1)	79.6 (76.2-83.1)	0.98 (0.91-1.04)	.83	73.8 (69.7-77.9)	0.91 (0.84-0.98)	.04
Overall	84.5 (83.9-85.1)	81.6 (81.0-82.3)	0.96 (0.95-0.97)	NA	82.9 (82.3-83.6)	0.98 (0.97-0.99)	NA
**Delayed medical care due to cost in past year**							
Asian	3.6 (2.5-4.6)	3.1 (2.2-4.0)	0.88 (0.57-1.34)	.93	4.4 (3.2-5.6)	1.25 (0.83-1.88)	.02
Black	10.5 (9.1-11.8)	7.0 (6.0-8.1)	0.68 (0.56-0.83)	.03	7.0 (5.9-8.1)	0.67 (0.54-0.82)	.22
Hispanic	13.2 (11.8-14.7)	8.7 (7.6-9.8)	0.66 (0.56-0.79)	.008	10.9 (9.7-12.2)	0.84 (0.72-0.97)	.32
White	8.1 (7.6-8.6)	7.0 (6.5-7.4)	0.86 (0.79-0.94)	[Reference]	6.2 (5.8-6.7)	0.77 (0.70-0.85)	[Reference]
Other^c^	11.6 (8.8-14.4)	11.0 (8.3-13.7)	0.94 (0.66-1.33)	.64	6.9 (4.5-9.3)	0.58 (0.38-0.87)	.17
Overall	9.1 (8.6-9.5)	7.1 (6.7-7.5)	0.82 (0.75-0.89)	NA	7.0 (6.6-7.4)	0.79 (0.73-0.87)	NA
**Did not receive medical care due to cost in past year**							
Asian	3.7 (2.3-5.0)	2.6 (1.8-3.4)	0.71 (0.44-1.15)	.71	3.8 (2.7-5.0)	1.07 (0.66-1.72)	.14
Black	11.1 (9.6-12.7)	7.3 (6.2-8.4)	0.66 (0.54-0.81)	.16	7.4 (6.1-8.6)	0.67 (0.54-0.82)	.40
Hispanic	12.2 (10.9-13.6)	8.0 (7.0-9.1)	0.66 (0.56-0.78)	.10	10.3 (9.1-11.5)	0.85 (0.73-0.99)	.10
White	7.1 (6.6-7.6)	5.5 (5.1-6.0)	0.78 (0.70-0.86)	[Reference]	5.2 (4.8-5.6)	0.74 (0.67-0.81)	[Reference]
Other^c^	9.6 (7.1-12.1)	8.9 (6.2-11.6)	0.92 (0.62-1.38)	.43	6.1 (4.1-8.1)	0.62 (0.40-0.95)	.43
Overall	8.3 (7.8-8.8)	6.1 (5.7-6.4)	0.75 (0.68-0.82)	NA	6.3 (5.9-6.7)	0.76 (0.69-0.83)	NA

Abbreviation: NA, not applicable.

^a^ Rate ratios are adjusted for age and sex. The reference group included 2019 data from White adults.

^b^ *P* value for interaction indicates whether there was a significant interaction between race and ethnicity × year compared with White adults.

^c^ Includes American Indian or Alaska Native, Native Hawaiian or other Pacific Islander, or multiple races or ethnicities.

^d^ Wellness visits include wellness visits, physical examinations, or general-purpose checkups.

### Cardiovascular Risk Factor Screening

**Figure 1.**
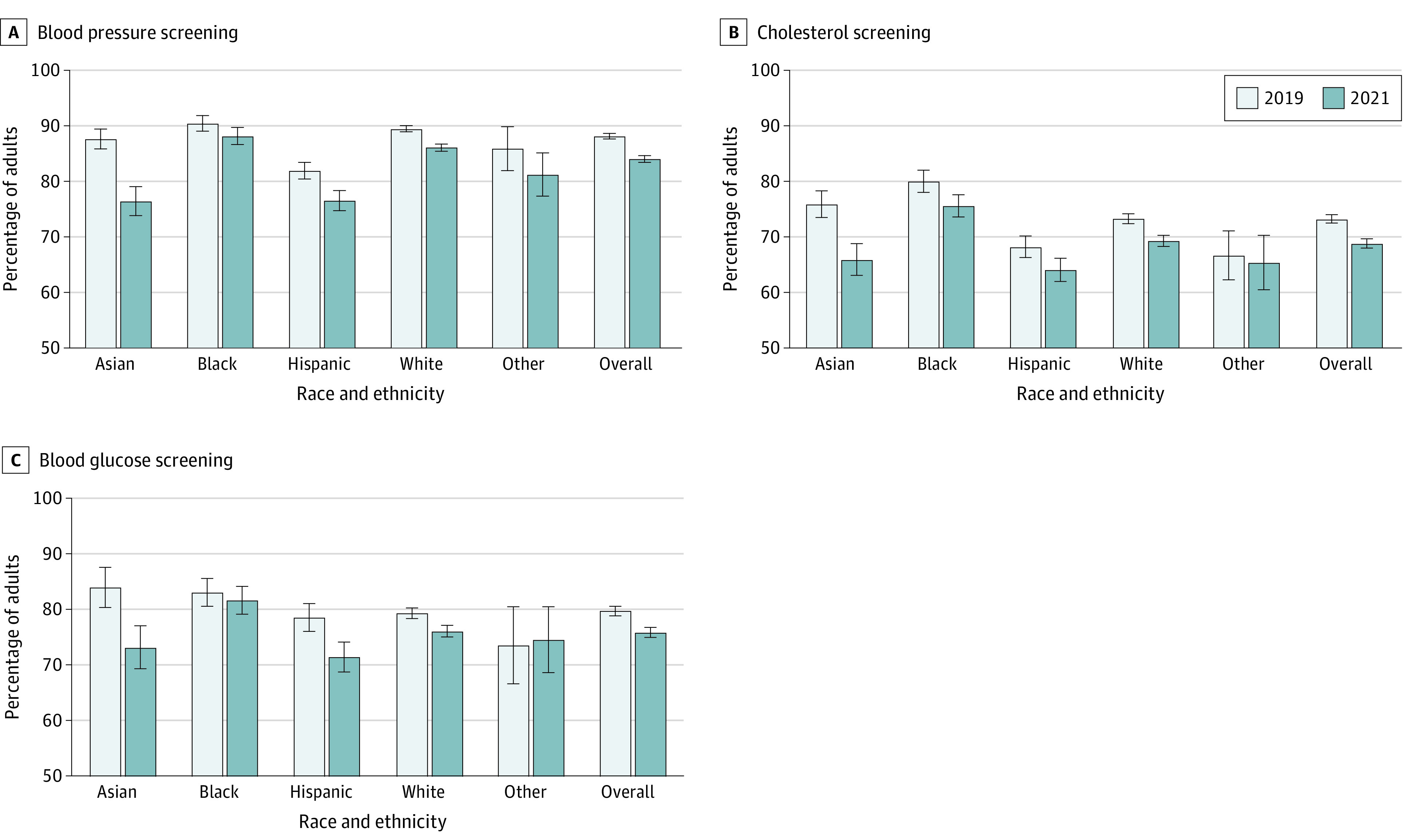
Proportion of Eligible US Adults Receiving Cardiovascular Risk Factor Screenings, 2019 and 2021 National estimates of the weighted proportion of eligible US adults receiving cardiovascular risk factor screening, both overall and by self-reported race and ethnicity. Other race and ethnicity includes American Indian or Alaska Native, Native Hawaiian or other Pacific Islander, or multiple races or ethnicities. All adults were considered eligible to receive blood pressure (A) and cholesterol screenings (B). Adults aged 45 years or older who have not previously been told they have diabetes were considered eligible to receive blood glucose screening (C). Error bars represent 95% CIs.

National estimates of the proportion of eligible adults who received cardiovascular risk factor screening are shown in Figure 1 and weighted screening rates and adjusted rate ratios are presented in eTable 1 in Supplement 1. After adjustment for age and sex, blood pressure (ARR, 0.95; 95% CI, 0.94-0.96) and cholesterol screening rates (ARR, 0.93; 95% CI, 0.92-0.94) in 2021 remained below 2019 levels, and these patterns were similar across all racial and ethnic subpopulations. Blood glucose screening rates were also lower in 2021 (compared with 2019) for the overall population (ARR, 0.95; 95% CI, 0.93-0.96) and for White, Asian, and Hispanic adults. The decline in the use of cardiovascular-related preventive services was largest for Asian adults (blood pressure screening: ARR, 0.87 [95% CI, 0.84-0.90]; cholesterol screening: ARR, 0.86 [95% CI, 0.82-0.91]; blood glucose screening: ARR, 0.87 [95% CI, 0.81-0.93]) when compared with White adults (*P* for interaction < .001 for blood pressure and cholesterol screenings and *P* for interaction < .01 for blood glucose screening).

### Cancer Screenings

**Figure 2.**
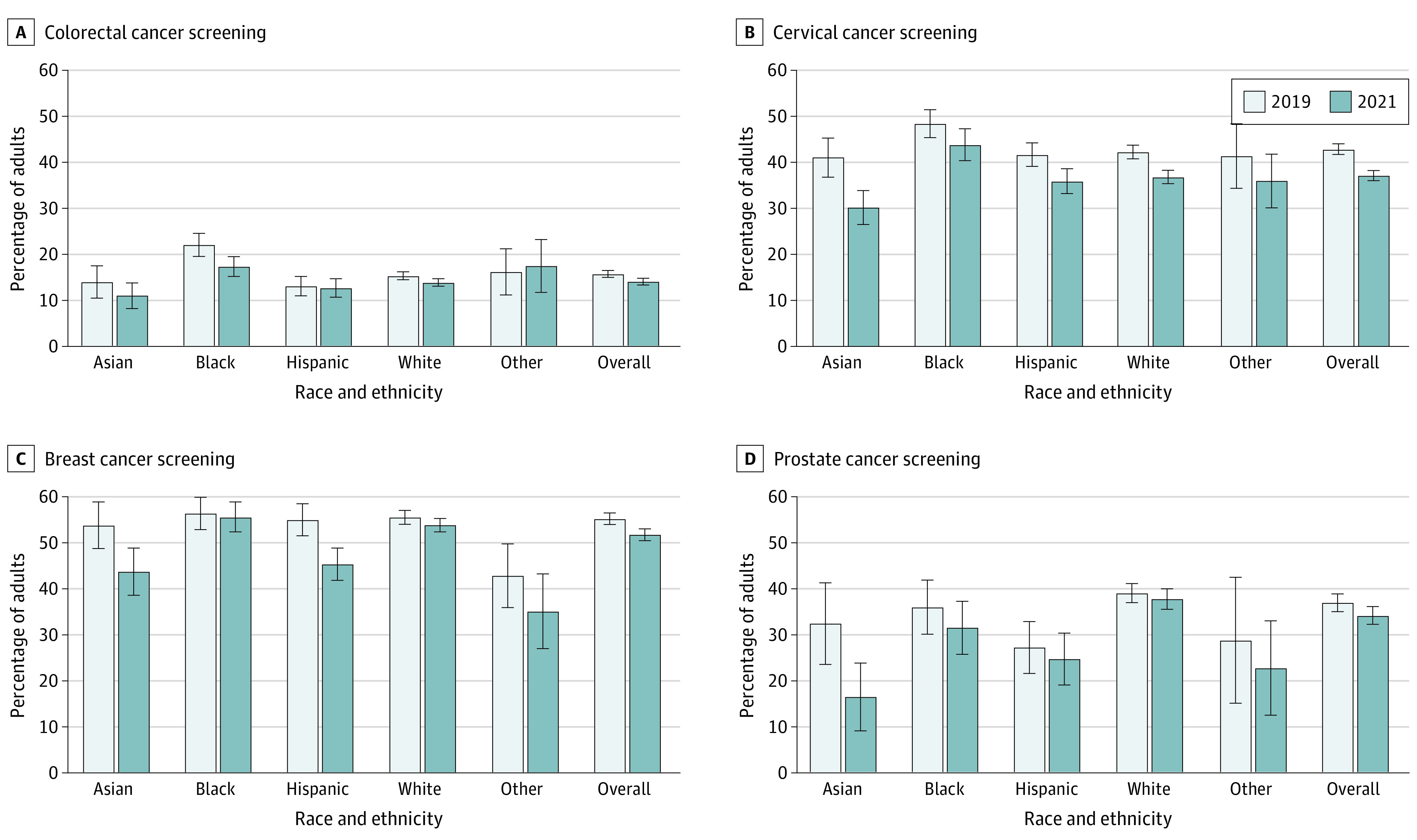
Proportion of Eligible US Adults Receiving Cancer Screenings, 2019 and 2021 National estimates of the weighted proportion of eligible US adults receiving cancer screening, both overall and by self-reported race and ethnicity. Other race and ethnicity includes American Indian or Alaska Native, Native Hawaiian or other Pacific Islander, and multiple races or ethnicities. Adults aged 50 to 75 years were considered eligible to receive colorectal cancer screening, consisting of colonoscopy or sigmoidoscopy (A). Female adults aged 21 to 65 years were considered eligible to receive cervical cancer screening, consisting of a Papanicolaou smear or human papillomavirus test (B). Female adults aged 50 to 74 years were considered eligible to receive breast cancer screening, consisting of mammography (C). Male adults aged 55 to 69 years were considered eligible to receive prostate cancer screening, consisting of a prostate-specific antigen test (D). Error bars represent 95% CIs.

**Figure 3.**
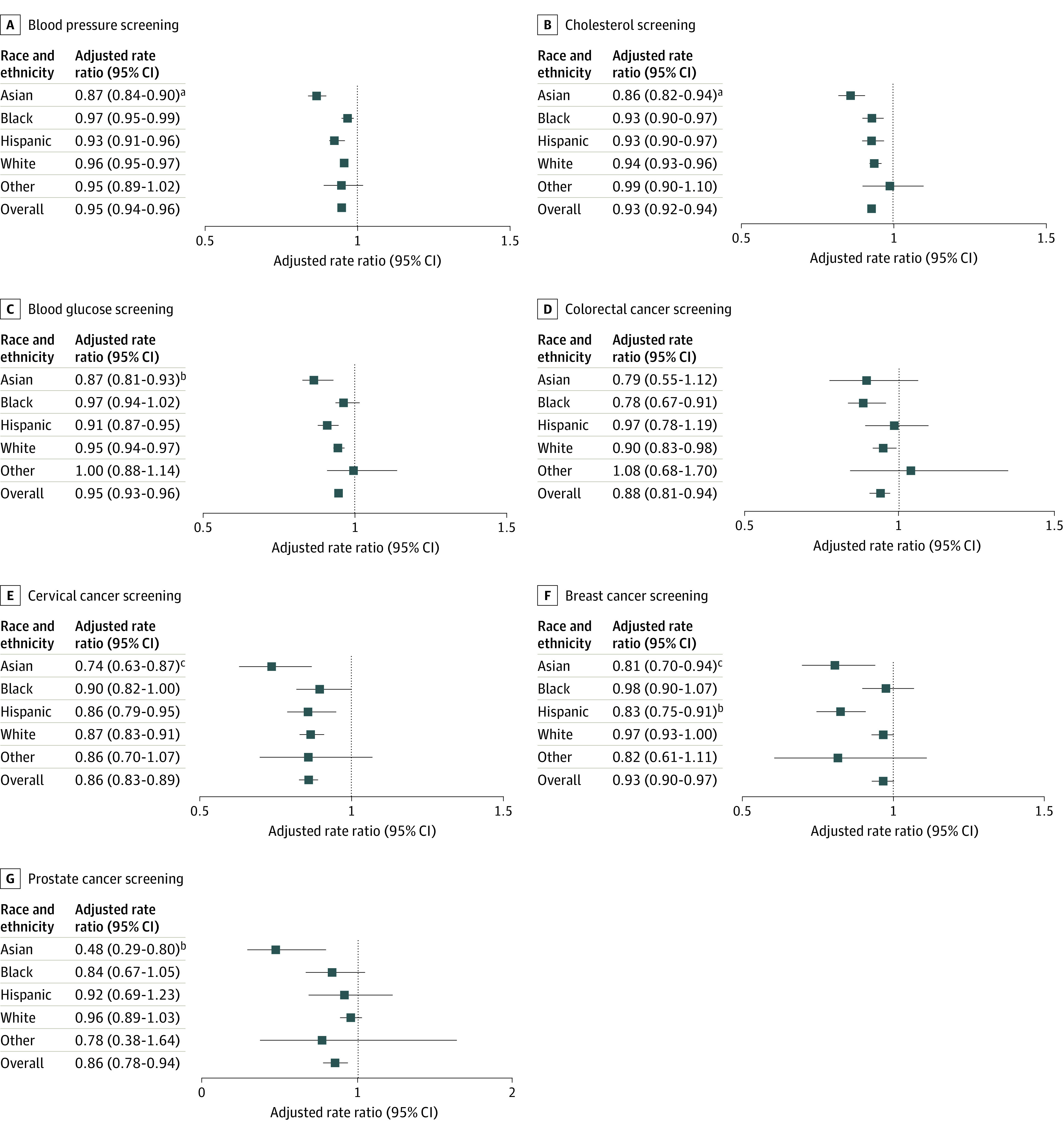
Age- and Sex-Adjusted Rate Ratios of Receiving Preventive Health Screenings Among Eligible Adults, 2021 vs 2019 Age- and sex-adjusted outcomes among US adults, both overall and by self-reported race and ethnicity. For each racial and ethnic group, the rate ratio represents outcomes in 2021 compared with 2019 (reference group). Other race and ethnicity includes American Indian or Alaska Native, Native Hawaiian or other Pacific Islander, or multiple races or ethnicities. All adults were considered eligible to receive blood pressure (A) and cholesterol screenings (B). Adults aged 45 years and older who have not previously been told they have diabetes were considered eligible to receive blood glucose screening (C). Adults aged 50 to 75 years were considered eligible to receive colorectal cancer screening, consisting of colonoscopy or sigmoidoscopy (D). Female adults aged 21 to 65 years were considered eligible to receive cervical cancer screening, consisting of a Papanicolaou smear or human papillomavirus test (E). Female adults aged 50 to 74 years were considered eligible to receive breast cancer screening, consisting of mammography (F). Male adults aged 55 to 69 years were considered eligible to receive prostate cancer screening, consisting of a prostate-specific antigen test (G). ^a^*P* for interaction < .001. ^b^*P* for interaction < .01. ^c^*P* for interaction < .05.

The proportion of adults who received cancer screening for each year are shown in Figure 2 and eTable 1 in Supplement 1. After adjustment for age and sex, eligible adults were less likely to receive colorectal cancer screening in 2021 compared with 2019 (ARR, 0.88; 95% CI, 0.81-0.94) (Figure 3). This decrease occurred primarily among Black adults (ARR, 0.78 [95% CI, 0.67-0.91]; *P* for interaction = .10) and White adults (ARR, 0.90; 95% CI, 0.83-0.98). Among eligible women, the cervical cancer screening rate was also lower in 2021 (ARR, 0.86; 95% CI, 0.83-0.89), a decline most pronounced for Asian women (ARR, 0.74 [95% CI, 0.63-0.87]; *P* for interaction = .05). Eligible women were also less likely to receive breast cancer screening in 2021 compared with 2019 (ARR, 0.93; 95% CI, 0.90-0.97), with the most pronounced decreases occurring among Asian (ARR, 0.81 [95% CI, 0.70-0.94]; *P* for interaction = .02) and Hispanic women (ARR, 0.83 [95% CI, 0.75-0.91]; *P* for interaction = .002). Among eligible men, the prostate cancer screening rate was lower in 2021 than in 2019 (ARR, 0.86; 95% CI, 0.78-0.94). When stratified by race and ethnicity, only Asian adults experienced a significant decline in prostate cancer screening in 2021 compared with 2019 (ARR, 0.48 [95% CI, 0.29-0.80]; *P* for interaction = .009).

### Additional Analyses

We performed a few additional analyses. First, the inclusion of socioeconomic factors (income, employment status, and insurance coverage) in the main models did not change associations between year and preventive health screenings (eTable 1 in Supplement 1). There were still significant declines in cardiovascular risk factor and cancer screenings in 2021 compared with 2019. Second, for preventive screenings, fewer adults were up to date on annual blood pressure screenings (ARR, 0.95; 95% CI, 0.94-0.96), but similar proportions of adults were up to date on their cholesterol, blood glucose, and cancer screenings in 2021 compared with 2019 (eTable 2 in Supplement 1). These patterns were similar in the overall US population and by race and ethnicity.

## Discussion

In this nationally representative study of US adults, we found that there were fewer outpatient wellness visits in 2021 and 2022 compared with prepandemic levels (2019), although cost-related barriers to accessing health care improved. In addition, the proportions of adults who received preventive health screenings were lower in 2021 compared with 2019, with significant variation by race and ethnicity. For example, Asian adults had the most pronounced decline in many preventive screenings, while Black and Hispanic adults had large declines in colorectal cancer and breast cancer screening, respectively. Differences in measures of health care access and preventive screenings between 2021 and 2022 compared with 2019 persisted even after adjusting for changes in income, employment status, and insurance coverage.

Our finding that preventive health screenings in 2021 did not return to prepandemic levels has major public health implications. This study builds on prior work by showing that the proportion of adults who had wellness visits remained below prepandemic levels through 2022—despite lower proportions of adults delaying or not seeking medical care due to costs—and that disruptions in cardiovascular risk factor screenings for the overall adult population, including those aged 18 to 40 years, persisted in 2021.^1,2,3,10,11,12^ Heart disease and cancer are leading causes of death in the US, and persistently lower rates of screenings for cardiovascular risk factors and cancer could have potentially devastating consequences for morbidity and mortality long term,^23,24,25,26^ particularly in light of rising cardiometabolic disease rates in young adults.^27,28^ Given that we found racial and ethnic minority populations received the fewest preventive screenings in 2019, a slower recovery from disruptions in these services during the pandemic may worsen health care disparities in future years. These findings highlight the urgent need for concerted health system, public health, and health policy efforts to increase preventive screenings among eligible US adults.

A few potential mechanisms might explain these overall declines in wellness visits and preventive screenings. First, fears of exposure to SARS-CoV-2 and other pathogens may have continued to deter people from attending in-person wellness visits through 2022.^29,30,31^ Second, care deferrals during the start of the COVID-19 public health emergency may have resulted in increased demand for wellness visits, and thus reduced availability. Appointment availability issues may have been further exacerbated by unclear clinic policies as practices gradually resumed wellness visits. Third, use of telemedicine remains above prepandemic levels.^32^ While telemedicine offers unique advantages—including patient convenience, rural access, and lower costs^33^—it is also associated with a decrease in preventive screenings because telemedicine often serves as a substitute for in-person visits and requires additional in-person laboratory visits to obtain many screening tests.^7^

Although there was initial concern that decreases in preventive health screenings may have been driven by loss of insurance coverage, unemployment, or economic loss, we found that changes during the pandemic persisted even after adjusting for these social factors. Moreover, our results suggest that financial barriers to receiving medical care decreased in 2021 and 2022 compared with 2019. This is consistent with our finding that income levels and health insurance coverage remained stable during the pandemic despite increases in unemployment likely due to state and federal efforts to bolster safety-net services (eg, Medicaid enrollment, supplemental income) as part of the COVID-19 public health emergency.^34^

We also observed significant differences in preventive screenings by race and ethnicity. For example, Asian adults experienced the most pronounced declines in cardiovascular risk factor screenings and many cancer screenings. This is concerning given that studies identified lower baseline rates of screenings among the overall Asian adult population and specific Asian subpopulations (ie, Chinese, Filipino, Indian, Korean, and Vietnamese adults) prior to the COVID-19 pandemic.^35,36,37^ Suggested explanations for these patterns have included the implications of diverse languages and dialects, stigmatized cultural perceptions of cancer, and use of complementary medicine.^35,38^ Furthermore, while medical financial concerns decreased in 2021 and 2022 for most racial and ethnic subpopulations, such concerns were similar across years for Asian adults. We also found that Black and Hispanic adults experienced large declines in colorectal and breast cancer screenings, respectively, compared with White adults. Given our finding that changes in insurance coverage, employment status, and income did not explain the large decline observed in these groups, other potential explanations could include other social factors that were not captured in the present study, such as lower availability of care in the patient’s preferred language or with culturally concordant clinicians, lower geographic proximity to primary care centers, or increased avoidance of in-person care (for reasons such as experienced racial discrimination) compared with White adults.^31,39^

Moving forward, an estimated 8 million to 24 million people are expected to lose Medicaid coverage by May 2024 as states discontinue Medicaid continuous enrollment with the end of the COVID-19 public health emergency.^40^ Given that Black and Hispanic adults, as well as some Asian subgroups, are more likely to receive coverage through Medicaid compared with White adults,^41^ loss of Medicaid coverage may exacerbate declines in wellness visits and preventive screenings in future years. Policy strategies to mitigate loss of Medicaid coverage are needed, and should be paired with efforts to launch and scale up culturally tailored, national quality improvement programs that use patient reminders, media campaigns, at-home tests, and community health center networks.^13,42,43,44,45,46,47^

### Limitations

This study has limitations. First, the response rate for the NHIS ranged from 50% to 60% across years. We used NHIS sampling weights to generate nationally representative estimates, which accounted for nonresponse bias.^15,16,17^Second, survey data are self-reported and may be subject to recall bias. However, it is unlikely recall bias differentially impacted responses between 2019 and 2021. Additionally, several studies^48,49^ have found the NHIS is a reliable measure of key health indicators, suggesting a strong concordance with results of other national surveys. Third, we were unable to further stratify the study group by race and ethnicity beyond the options predefined by the NHIS. Heterogeneity exists within each of the studied racial and ethnic subpopulations and additional research should be conducted to identify potential differences in preventive screening within these subpopulations to properly direct resources and tailor interventions. Fourth, we did not include at-home colorectal cancer screening tests in our analysis due to different survey coding and branching logic between years, likely underestimating the true rate of colorectal cancer screenings. However, colonoscopies and sigmoidoscopies represented over 60% of colorectal cancer screening tests received as of 2021.^50^ Finally, NHIS 2023 data were not available at the time of this analysis, and future research should continue to monitor changes in health care access and preventive screenings as the US emerges from the pandemic.

## Conclusion

In this cross-sectional study of nationally representative data, outpatient wellness visits among US adults in 2022 remained below prepandemic levels, although cost-related barriers to accessing health care improved since 2019. In addition, preventive health screenings were lower in 2021 compared with 2019, with the most pronounced declines occurring among racial and ethnic minority groups. These findings were not explained by pandemic-related changes in socioeconomic factors (income, employment status, insurance coverage). Targeted public health efforts are needed to increase preventive health screenings among eligible adults as the US emerges from the pandemic.

## Notes

Supplement 1. **eTable 1.** Age-, Sex-, and Socioeconomic-Adjusted Rate Ratios of Receiving Preventive Health Screenings Among Eligible US Adults, 2021 versus 2019 **eTable 2.** Age- and Sex-Adjusted Rate Ratios of Being Up to Date on Preventive Health Screenings Among Eligible US Adults, 2021 versus 2019

Supplement 2.
Data Sharing Statement
