# Supplementary material for: Changes in Health Care Access and Preventive Health Screenings by Race and Ethnicity
Source: JAMA Health Forum. 2024 Feb 2;5(2):e235058. doi: 10.1001/jamahealthforum.2023.5058 (PMC10837752; doi:10.1001/jamahealthforum.2023.5058)
Supplement: Supplement 1. — eTable 1. Age-, Sex-, and Socioeconomic-Adjusted Rate Ratios of Receiving Preventive Health Screenings Among Eligible US Adults, 2021 versus 2019 eTable 2. Age- and Sex-Adjusted Rate Ratios of Being Up to Date on Preventive Health Screenings Among Eligible US Adults, 2021 versus 2019 [file jamahealthforum-e235058-s001.pdf]

Supplemental Online Content

Alba C, Zheng Z, Wadhera RK. Changes in health care access and preventive health screenings by race and ethnicity. *JAMA Health Forum.* 2024;5(2):e235058. doi:10.1001/jamahealthforum.2023.5058

eTable 1. Age-, Sex-, and Socioeconomic-Adjusted Rate Ratios of Receiving Preventive Health Screenings Among Eligible US Adults, 2021 versus 2019

eTable 2. Age- and Sex-Adjusted Rate Ratios of Being Up to Date on Preventive Health Screenings Among Eligible US Adults, 2021 versus 2019

This supplemental material has been provided by the authors to give readers additional information about their work.

eTable 1. Age-, Sex-, and Socioeconomic-Adjusted Rate Ratios of Receiving Preventive Health Screenings Among Eligible US Adults, 2021 versus 2019

	2019 (weighted rate) [a]	2021 (weighted rate)[a]	Age- and sex-adjusted rate ratio, 95% CI[b] *(2021 vs 2019)*	P-value for interaction[c]	Age-, sex-, and socioeconomic-adjusted rate ratio, 95% CI[d] *(2021 vs 2019)*	P-value for interaction[c]
Blood pressure checked in past year						
White	89.4 (88.9, 90.0)	86.1 (85.4, 86.7)	0.96 (0.95, 0.97)	Reference	0.96 (0.95, 0.96)	Reference
Asian	87.6 (85.8, 89.4)	76.4 (73.8, 79.0)	0.87 (0.84, 0.90)	<0.001	0.86 (0.83, 0.89)	<0.001
Black	90.4 (89.0, 91.8)	88.1 (86.6, 89.7)	0.97 (0.95, 0.99)	0.40	0.96 (0.94, 0.99)	0.47
Hispanic	81.9 (80.4, 83.4)	76.5 (74.7, 78.3)	0.93 (0.91, 0.96)	0.05	0.93 (0.90, 0.95)	0.03
Other[e]	85.9 (81.9, 89.8)	81.2 (77.3, 85.1)	0.95 (0.89, 1.02)	0.77	0.94 (0.88, 1.01)	0.65
Overall	88.1 (87.6, 88.6)	84.0 (83.4, 84.6)	0.95 (0.94, 0.96)	NA	0.94 (0.94, 0.95)	NA
Cholesterol checked in past year						
White	73.3 (72.4, 74.2)	69.3 (68.3, 70.3)	0.94 (0.93, 0.96)	Reference	0.93 (0.92, 0.95)	Reference
Asian	75.9 (73.5, 78.3)	65.9 (63.1, 68.8)	0.86 (0.82, 0.91)	<0.001	0.85 (0.81, 0.90)	<0.001
Black	80.0 (78.0, 82.0)	75.6 (73.6, 77.6)	0.93 (0.90, 0.97)	0.72	0.93 (0.90, 0.96)	0.84
Hispanic	68.2 (66.3, 70.2)	64.1 (62.0, 66.2)	0.93 (0.90, 0.97)	0.70	0.93 (0.89, 0.96)	0.80
Other[e]	66.7 (62.3, 71.1)	65.4 (60.5, 70.3)	0.99 (0.90, 1.10)	0.29	0.99 (0.90, 1.09)	0.22
Overall	73.2 (72.5, 74.0)	68.8 (68.0, 69.7)	0.93 (0.92, 0.94)	NA	0.92 (0.91, 0.94)	NA
Blood sugar test in last year[f]						
White	79.4 (78.4, 80.3)	76.1 (75.1, 77.2)	0.95 (0.94, 0.97)	Reference	0.95 (0.93, 0.96)	Reference
Asian	84.0 (80.4, 87.6)	73.2 (69.4, 77.1)	0.87 (0.81, 0.93)	0.01	0.85 (0.79, 0.91)	0.003
Black	83.1 (80.6, 85.6)	81.7 (79.2, 84.2)	0.97 (0.94, 1.02)	0.33	0.97 (0.94, 1.01)	0.19
Hispanic	78.6 (76.1, 81.1)	71.5 (68.8, 74.2)	0.91 (0.87, 0.95)	0.06	0.91 (0.87, 0.95)	0.09
Other[e]	73.6 (66.7, 80.5)	74.6 (68.7, 80.5)	1.00 (0.88, 1.14)	0.43	0.99 (0.88, 1.12)	0.43
Overall	79.8 (78.9, 80.6)	75.9 (75.0, 76.8)	0.95 (0.93, 0.96)	NA	0.94 (0.92, 0.95)	NA
Received a colonoscopy or sigmoidoscopy in last year[g]						
White	15.1 (14.3, 16.0)	13.7 (12.9, 14.5)	0.90 (0.83, 0.98)	Reference	0.88 (0.81, 0.96)	Reference
Asian	13.8 (10.3, 17.3)	10.9 (8.1, 13.6)	0.79 (0.55, 1.12)	0.47	0.73 (0.52, 1.04)	0.31
Black	21.9 (19.4, 24.4)	17.2 (15.0, 19.3)	0.78 (0.67, 0.91)	0.10	0.79 (0.67, 0.92)	0.20

	2019 (weighted rate) [a]	2021 (weighted rate)[a]	Age- and sex-adjusted rate ratio, 95% CI[b] *(2021 vs 2019)*	P-value for interaction[c]	Age-, sex-, and socioeconomic-adjusted rate ratio, 95% CI[d] *(2021 vs 2019)*	P-value for interaction[c]
Hispanic	12.9 (10.8, 15.0)	12.5 (10.5, 14.5)	0.97 (0.78, 1.19)	0.56	0.93 (0.75, 1.15)	0.69
Other[e]	16.0 (11.0, 21.0)	17.3 (11.6, 23.0)	1.08 (0.68, 1.70)	0.45	1.10 (0.68, 1.76)	0.38
Overall	15.5 (14.8, 16.3)	13.9 (13.2, 14.6)	0.88 (0.81, 0.94)	NA	0.85 (0.8, 0.92)	NA
Received cervical cancer screening in last year[h]						
White	42.2 (40.8, 43.7)	36.8 (35.4, 38.3)	0.87 (0.83, 0.91)	Reference	0.86 (0.82, 0.90)	Reference
Asian	41.1 (36.8, 45.3)	30.2 (26.5, 33.9)	0.74 (0.63, 0.87)	0.05	0.74 (0.63, 0.86)	0.06
Black	48.4 (45.4, 51.4)	43.8 (40.4, 47.3)	0.90 (0.82, 1.00)	0.50	0.90 (0.82, 1.00)	0.42
Hispanic	41.6 (39.1, 44.2)	35.9 (33.2, 38.6)	0.86 (0.79, 0.95)	0.84	0.87 (0.8, 0.96)	0.79
Other[e]	41.4 (34.4, 48.4)	36.0 (30.1, 41.8)	0.86 (0.70, 1.07)	0.94	0.85 (0.69, 1.04)	0.88
Overall	42.8 (41.7, 44.0)	37.1 (36.0, 38.2)	0.86 (0.83, 0.89)	NA	0.85 (0.82, 0.89)	NA
Received mammogram in last year[i]						
White	55.3 (53.8, 56.8)	53.6 (52.1, 55.0)	0.97 (0.93, 1.00)	Reference	0.96 (0.92, 0.99)	Reference
Asian	53.5 (48.5, 58.6)	43.5 (38.4, 48.6)	0.81 (0.7, 0.94)	0.02	0.79 (0.68, 0.91)	0.01
Black	56.1 (52.6, 59.6)	55.3 (52.1, 58.6)	0.98 (0.9, 1.07)	0.80	0.95 (0.87, 1.04)	0.93
Hispanic	54.7 (51.3, 58.2)	45.1 (41.6, 48.6)	0.83 (0.75, 0.91)	0.002	0.81 (0.74, 0.9)	0.002
Other[e]	42.6 (35.7, 49.5)	34.9 (26.8, 43.0)	0.82 (0.61, 1.11)	0.29	0.80 (0.60, 1.06)	0.22
Overall	54.9 (53.7, 56.2)	51.5 (50.2, 52.8)	0.93 (0.90, 0.97)	NA	0.92 (0.89, 0.95)	NA
Received a PSA test in last year[j]						
White	38.8 (36.7, 40.9)	37.5 (35.3, 39.7)	0.96 (0.89, 1.03)	Reference	0.95 (0.88, 1.02)	Reference
Asian	32.2 (23.3, 41.0)	16.3 (8.9, 23.6)	0.48 (0.29, 0.80)	0.009	0.52 (0.31, 0.85)	0.02
Black	35.7 (29.9, 41.6)	31.3 (25.5, 37.0)	0.84 (0.67, 1.05)	0.28	0.81 (0.64, 1.01)	0.18
Hispanic	27.0 (21.4, 32.6)	24.5 (18.9, 30.1)	0.92 (0.69, 1.23)	0.81	0.94 (0.70, 1.26)	0.97
Other[e]	28.5 (14.9, 42.2)	22.5 (12.3, 32.8)	0.78 (0.38, 1.64)	0.59	0.72 (0.38, 1.34)	0.39
Overall	36.7 (34.8, 38.6)	33.9 (32.0, 35.9)	0.86 (0.78, 0.94)	NA	0.85 (0.77, 0.93)	NA

Abbreviations: CI, confidence interval; FOBT, fecal occult blood test; FIT, fecal immunochemical test; PSA, prostate-specific antigen; NA, not applicable.

[a] Nationally representative (%) estimates are shown with 95% confidence intervals.

[b] Multivariable models adjusted for age and sex. The reference group was 2019.
[c] The interaction p-value indicates whether there was a significant change in outcome (2021 versus 2019) for each racial and ethnic minority subgroup compared with non-Hispanic White adults.
[d] Multivariable models adjusted for age, sex, income, employment status, and insurance coverage. The reference group was 2019.
[e] Other includes people who identified as American Indian, Alaskan Native, Other, or multiple race/ethnicities, including Native Hawaiian or Pacific Islander.
[f] Only among those who have not previously been told they have diabetes and are 45+ years old.
[g] Only among those who are 50-75 years old.
[h] Among all female respondents who are 21-65 years old. Cervical cancer screening includes those who reported receiving a pap smear and/or human papillomavirus test.
[i] Among all female respondents who are 50-74 years old.
[j] Among all male respondents who are 55-69 years old.

eTable 2. Age- and Sex-Adjusted Rate Ratios of Being Up to Date on Preventive Health Screenings Among Eligible US Adults, 2021 versus 2019

	2019 (weighted rate)[a]	2021 (weighted rate)[a]	Age- and sex-adjusted rate ratio, 95% CI[b] *(2021 vs 2019)*	P-value for interaction[c]	Age-, sex-, and socioeconomic-adjusted rate ratio, 95% CI[d] *(2021 vs 2019)*	P-value for interaction[c]
Blood pressure checked in past year[e]						
White	89.4 (88.9, 90.0)	86.1 (85.4, 86.7)	0.96 (0.95, 0.97)	Reference	0.96 (0.95, 0.96)	Reference
Asian	87.6 (85.8, 89.4)	76.4 (73.8, 79.0)	0.87 (0.84, 0.90)	<0.001	0.86 (0.83, 0.89)	<0.001
Black	90.4 (89.0, 91.8)	88.1 (86.6, 89.7)	0.97 (0.95, 0.99)	0.40	0.96 (0.94, 0.99)	0.47
Hispanic	81.9 (80.4, 83.4)	76.5 (74.7, 78.3)	0.93 (0.91, 0.96)	0.05	0.93 (0.90, 0.95)	0.03
Other[f]	85.9 (81.9, 89.8)	81.2 (77.3, 85.1)	0.95 (0.89, 1.02)	0.77	0.94 (0.88, 1.01)	0.64
Overall	88.1 (87.6, 88.6)	84.0 (83.4, 84.6)	0.95 (0.94, 0.96)	NA	0.94 (0.94, 0.95)	NA
Cholesterol checked in past 5 years[e]						
White	90.8 (90.2, 91.4)	90.9 (90.2, 91.6)	1.00 (0.99, 1.01)	Reference	1.00 (0.99, 1.00)	Reference
Asian	90.4 (88.6, 92.1)	92.7 (91.2, 94.3)	1.02 (1.00, 1.05)	0.08	1.02 (1.00, 1.05)	0.06
Black	93.1 (91.8, 94.4)	93.1 (91.9, 94.3)	1.00 (0.98, 1.01)	0.66	1.00 (0.98, 1.01)	0.90
Hispanic	88.3 (86.8, 89.7)	89.3 (87.9, 90.7)	1.01 (0.99, 1.03)	0.38	1.01 (0.99, 1.03)	0.26
Other[f]	85.9 (82.5, 89.3)	87.8 (83.9, 91.7)	1.03 (0.97, 1.09)	0.37	1.03 (0.97, 1.09)	0.30
Overall	90.5 (90.0, 91.0)	90.9 (90.3, 91.5)	1.00 (1.00, 1.01)	NA	1.00 (0.99, 1.01)	NA
Blood sugar test in last 3 years[g]						
White	89.0 (88.3, 89.8)	89.0 (88.3, 89.7)	1.00 (0.99, 1.01)	Reference	1.00 (0.98, 1.01)	Reference
Asian	90.6 (87.9, 93.4)	90.9 (88.6, 93.2)	1.00 (0.96, 1.04)	0.80	0.99 (0.96, 1.03)	0.99
Black	91.3 (89.7, 92.9)	92.0 (90.5, 93.5)	1.01 (0.98, 1.03)	0.59	1.00 (0.98, 1.02)	0.70
Hispanic	90.1 (88.4, 91.8)	89.7 (88.1, 91.3)	1.00 (0.97, 1.02)	0.85	0.99 (0.97, 1.02)	0.94
Other[f]	87.2 (83.6, 90.7)	89.0 (85.5, 92.5)	1.02 (0.96, 1.08)	0.46	1.01 (0.96, 1.06)	0.63
Overall	89.5 (88.8, 90.1)	89.5 (88.9, 90.1)	1.00 (0.99, 1.01)	NA	1.00 (0.99, 1.00)	NA
Received a colonoscopy in last 10 years or sigmoidoscopy in last 5 years[h]						
White	66.3 (65.2, 67.5)	66.9 (65.8, 68.0)	1.00 (0.98, 1.02)	Reference	0.99 (0.97, 1.01)	Reference
Asian	51.3 (45.9, 56.8)	50.9 (46.1, 55.7)	1.00 (0.87, 1.14)	0.96	0.99 (0.86, 1.13)	0.94
Black	64.0 (61.1, 66.8)	63.8 (60.7, 66.8)	1.00 (0.94, 1.06)	0.99	1.00 (0.94, 1.06)	0.45

	2019 (weighted rate)[a]	2021 (weighted rate)[a]	Age- and sex-adjusted rate ratio, 95% CI[b] *(2021 vs 2019)*	P-value for interaction[c]	Age-, sex-, and socioeconomic-adjusted rate ratio, 95% CI[d] *(2021 vs 2019)*	P-value for interaction[c]
Hispanic	46.3 (43.0, 49.6)	51.1 (47.9, 54.4)	1.1 (1.01, 1.19)	0.4	1.06 (0.98, 1.16)	0.09
Other[f]	56.3 (48.9, 63.6)	54.5 (47.6, 61.3)	0.96 (0.80, 1.16)	0.71	0.94 (0.75, 1.17)	0.66
Overall	62.7 (61.6, 63.7)	63.5 (62.5, 64.6)	1.00 (0.98, 1.03)	NA	0.99 (0.97, 1.02)	NA
Received cervical cancer screening in last 3 or 5 years[i]						
White	69.8 (68.4, 71.1)	69.7 (68.3, 71.0)	1.00 (0.97, 1.02)	Reference	0.99 (0.97, 1.02)	Reference
Asian	61.4 (56.9, 65.9)	57.1 (52.9, 61.3)	0.93 (0.84, 1.04)	0.24	0.94 (0.84, 1.04)	0.27
Black	69.8 (66.6, 73.0)	67.0 (63.6, 70.4)	0.96 (0.90, 1.02)	0.27	0.96 (0.90, 1.02)	0.30
Hispanic	64.8 (62.0, 67.7)	63.2 (60.4, 66.0)	0.97 (0.92, 1.04)	0.49	0.97 (0.92, 1.03)	0.51
Other[f]	65.7 (58.0, 73.3)	64.0 (56.4, 71.5)	0.97 (0.86, 1.10)	0.68	0.96 (0.85, 1.09)	0.65
Overall	68.2 (67.1, 69.4)	67.1 (65.9, 68.3)	0.98 (0.96, 1.01)	NA	0.98 (0.95, 1.00)	NA
Received mammogram in last 2 years[j]						
White	75.2 (73.7, 76.7)	75.1 (73.8, 76.5)	1.00 (0.97, 1.02)	Reference	0.99 (0.97, 1.01)	Reference
Asian	70.3 (63.5, 77.1)	64.9 (59.2, 70.6)	0.92 (0.81, 1.05)	0.24	0.90 (0.80, 1.02)	0.14
Black	76.5 (72.8, 80.3)	79.8 (76.5, 83.1)	1.04 (0.98, 1.11)	0.23	1.04 (0.97, 1.11)	0.20
Hispanic	77.0 (73.1, 81.0)	71.9 (68.3, 75.5)	0.93 (0.87, 1.00)	0.08	0.92 (0.86, 0.98)	0.04
Other[f]	67.4 (59.1, 75.8)	62.8 (54.3, 71.3)	0.93 (0.78, 1.12)	0.46	0.96 (0.81, 1.15)	0.77
Overall	75.2 (73.9, 76.4)	74.5 (73.3, 75.7)	0.99 (0.96, 1.02)	NA	0.98 (0.96, 1.01)	NA
Received a PSA test in last 2 years[k]						
White	47.1 (45.0, 49.3)	46.3 (44.1, 48.5)	0.97 (0.91, 1.04)	Reference	0.96 (0.90, 1.02)	Reference
Asian	38.4 (28.9, 47.8)	28.4 (19.4, 37.3)	0.71 (0.49, 1.02)	0.09	0.76 (0.53, 1.07)	0.18
Black	45.0 (39.0, 51.0)	42.5 (36.2, 48.8)	0.91 (0.76, 1.10)	0.51	0.88 (0.74, 1.06)	0.38
Hispanic	38.5 (31.9, 45.0)	33.3 (27.4, 39.3)	0.88 (0.69, 1.12)	0.43	0.88 (0.69, 1.13)	0.52
Other[f]	30.2 (16.4, 44.0)	28.8 (16.5, 41.0)	0.95 (0.47, 1.89)	0.94	0.86 (0.48, 1.53)	0.71
Overall	45.2 (43.3, 47.2)	43.1 (41.1, 45.1)	0.92 (0.85, 0.99)	NA	0.91 (0.85, 0.98)	NA

Abbreviations: CI, confidence interval; FOBT, fecal occult blood test; FIT, fecal immunochemical test; PSA, prostate-specific antigen; NA, not applicable.

[a] Nationally representative (%) estimates are shown with 95% confidence intervals.

[b] Multivariable models adjusted for age and sex. The reference group was 2019.
[c] The interaction p-value indicates whether there was a significant change in outcome (2021 versus 2019) for each racial and ethnic minority subgroup compared with non-Hispanic White adults.
[d] Multivariable models adjusted for age, sex, income, employment status, and insurance coverage. The reference group was 2019.
[e] Among all adults who are 18+ years old. The Centers for Disease Control and Prevention recommend a cholesterol screening every 4 to 6 years. For this analysis, an adult is considered up-to-date on cholesterol screening if they've received one in the past 5 years due to pre-specified survey responses on the National Health Interview Survey.
[f] Other includes people who identified as American Indian, Alaskan Native, Other, or multiple race/ethnicities, including Native Hawaiian or Pacific Islander.
[g] Only among those who have not previously been told they have diabetes and are 45+ years old.
[h] Only among those who are 50-75 years old.
[i] Among all female respondents who are 21-65 years old. A female respondent who is 21-29 years old is considered up-to-date on cervical cancer screening if they have received a pap smear in the past 3 years. A female respondent who is 30-65 years old is considered up-to-date on cervical cancer screening if they have received a pap smear alone in the past 3 years, HPV test alone in the past 5 years, or a pap smear with HPV test in the past 5 years.
[j] Among all female respondents who are 50-74 years old.
[k] Among all male respondents who are 55-69 years old.
