# Supplementary material for: Changes in Health Care Access and Preventive Health Screenings by Race and Ethnicity
Source: JAMA Health Forum. 2024 Feb 2;5(2):e235058. doi: 10.1001/jamahealthforum.2023.5058 (PMC10837752; doi:10.1001/jamahealthforum.2023.5058)
Supplement: Supplement 2. — Data Sharing Statement [file jamahealthforum-e235058-s002.pdf]

Data Sharing Statement

Alba. Changes in Health Care Access and Preventive Health Screenings by Race and Ethnicity. *JAMA Health Forum*. Published February 02, 2024.
doi:10.1001/jamahealthforum.2023.5058

Data
Data available: No

Additional Information
Explanation for why data not available: All data used for this study are publicly-available for free to researchers on the NHIS website
